# Parent Perspectives: Part 2—Considerations for the Transition Home Post-NICU Discharge

**DOI:** 10.3390/children10121835

**Published:** 2023-11-22

**Authors:** Jessica N. DiBari, LaToshia Rouse

**Affiliations:** 1 U.S. Department of Health and Human Services, Health Resources and Services Administration, Maternal and Child Health Bureau, Rockville, MD 20857, USA; 2 Certified Doula at Birth Sisters Doula Services and Patient Engagement Consultant, Knightdale, NC 27545, USA

**Keywords:** neonatal intensive care unit, preterm birth, life course, collaborative care models, family centered care, pediatric transitions, parent perspectives

## Abstract

This paper is part two of a series of papers written by the mothers of Neonatal Intensive Care Unit (NICU) graduates. The companion paper, “*Parent Perspectives: Part 1—Considerations for Changing the NICU Culture*”, considers all aspects of the NICU experience and provides recommendations for interventions and improvements from a life-course perspective while families are in the NICU. In part two, the focus is the transition home post-NICU stay. The time after NICU discharge is a critical and sensitive developmental period for NICU babies and their families, and an important life course transition. This paper provides a parent’s perspective of how to improve the transition home post-NICU stay. Our perspectives draw on the Life Course Health Development approach, which regards health as an active process that is developed over time based on a person’s internal biologic and physiologic systems, their external environment and circumstances, and the interactions or relationships between them. This paper describes a collaborative care model where parents and their healthcare teams work together to develop shared care plans. It also describes how we can build trust and family capacity to support long-term care, ensure family well-being, and link families to needed resources and support that can ease the transition from the NICU back to the home and optimize family health trajectories.

## 1. Introduction

This paper is part two of a series of papers written by mothers of NICU babies. The companion paper “*Parent Perspectives: Part 1—Considerations for Changing the NICU Culture*” considers all aspects of the NICU experience and provides recommendations for interventions and improvements from a life-course perspective. In this paper, the authors focus on the process of the transition home as a critical and sensitive developmental period for NICU babies and their families, and an important life-course transition. This paper draws on the Life Course Health Development approach, which regards health as an active process that is developed over time based on a person’s internal biologic and physiologic systems, their external environment and circumstances, and the interactions or relationships between them [1]. All aspects of the child’s environment (family, community, social and relational environment) support the child on a path to thriving during and after the critical transition from the NICU to the home [2]. The paper also highlights the critical importance of family well-being, both in terms of the ability of the family to buffer the infant from environmental risk factors, but also in terms of the family’s own health development trajectory that will be critical to supporting the developing child in the years to come [3]. Failure to support the family adequately while transitioning home could have major implications for the future health and well-being of the family and child, setting off a cumulative chain of risks resulting in long-term adverse consequences. Conversely, optimal support can set the child and family on the healthiest of possible life course trajectories, paying major dividends through to adult life and even impacting the next generation.

## 2. Partner with Families to Support the Transition to the Home

The transition to the home can be intimidating. The family may feel ill prepared in many ways. Prior to discharge from the NICU, every family must gain the skills necessary to independently care for their infant at home (e.g., CPR skills, administering medication, operating medical equipment, etc.). While these care needs vary considerably, even aspects of care regarded by medical staff as “minor and low-risk” can be intimidating and anxiety provoking for parents. Families no longer have the medical equipment that was available in the NICU to monitor vitals and medical staff are not readily available to step in. If families are to be supported adequately during this transition, it is essential that they form a partnership with the medical team. It is through these medical–family partnerships that families can build their capacity for caregiving and gain confidence for post-NICU care.

When preparing for the transition home, parents should be encouraged to participate in daily rounds at the hospital or virtually, provide physical care for their baby, learn the warning signs for when medical intervention is needed, and gain an understanding of what will be required after discharge. Families will participate at their comfort level. These interactions will help parents sharpen the skills needed post-discharge. In a true collaborative care model, where providers and families form a partnership to address the child’s care needs, parents may discuss the day’s concerns, ask questions, offer insights about their child, make recommendations, be involved in decision-making, and share their expectations. During rounds, it can also be intimidating to hear complex medical information being rapidly conveyed. Having parents speak first during rounds can ensure parents’ concerns are addressed before staff provide updates. Providing an opportunity for parents to voice their concerns can create a sense of belonging and reduce some of the hierarchy between parents and staff while helping families to build healthy literacy skills for post-NICU life. Involvement levels will vary and communication styles must be adaptive to effectively communicate with each family. Building confidence around care decisions and ensuring resources are available and accessible are essential. Families in the NICU build advocacy skills, caregiving skills and navigation skills that are needed in their community to help their baby thrive. Families will need to use these skills when communicating with providers and gathering community resources. Developing a collaborative care model in the NICU prepares families for future care collaborations, makes room for autonomy, builds rapport between the providers and family, reduces stress/anxiety, and ultimately can lead to better outcomes for the newborn.

## 3. Embrace a Collaborative Care Model

A collaborative care model that integrates family involvement is also beneficial for the child’s development. Many factors can impact parent participation in the NICU and not all families will be there as much as desired; however, this can be improved if families understand the benefits to their child at this early stage and beyond. Holding the child and skin-to-skin care can lead to better developmental outcomes [4]. This highlights the importance of engaging families in the NICU. A consistent collaborative care model can empower families. Parental involvement and learning at every opportunity can shift the culture of the NICU to allow for full parenting in a NICU setting and ease the transition home [5]. The nursing staff should encourage parents to change diapers, give baths, administer medication, flush a gastrointestinal tube (G-tube), feed with a syringe, remove tape from skin, etc. Providing supportive feedback and setting competency and comfort goals can help improve caregiver confidence prior to discharge. This can also be overwhelming for some parents so the staff should ask families if they are ready to learn these hands-on tasks. The willingness to be engaged may relate to the timing of being asked and a myriad of other factors. Ultimately, the level of engagement is at the parents’ discretion depending on their comfort level. Providers can suggest opportunities for parental engagement over time but also must be respectful if a parent has reservations.

## 4. Build Essential Skills

To the fullest extent possible, providers should exhibit safe sleep practices in the last few weeks before discharge; no tilted/inclined bed and nothing left in the crib. This will make the transition home easier on both the family and the baby. If families will have any special equipment at home, they need to be educated on how to use this equipment over time, not just 15 min prior to discharge. Tasks like replacing a G-tube, changing oxygen tanks, learning to care for a tracheostomy, and putting on a pulse ox should be taught with the exact equipment that will be used at home during discharge planning [6]. Online and in-person training courses before discharge can help with the educational needs of a family [7,8]. Some NICUs have car seat safety classes, G-tube care, SIDS education, and other learning resources. Recorded classes and/or written instructions that reflect the diversity of the NICU population could serve as tools to help families remember key steps as they begin to perform these tasks on their own and help to standardize instructions. These educational opportunities can also build confidence around caretaking post-discharge.

## 5. Overcome the “Fragile Baby” Mindset

Although most families long to have their baby at home, it can be intimidating to bring home a baby who was once supported by so many machines and tubes and had so many medical needs. Some families may be transitioning home with medical equipment which also may come with a steep learning curve to operate correctly. It helps to remind families that babies are not discharged until they are strong enough to go home. It is easy to slip into the mindset that the baby is fragile. Families will need support over the first few weeks as they transition into a new routine at home. Making sure families have a non-crisis support line to call if they have questions can ease the transition. Offering peer support from NICU families who have graduated can help families build community and benefit from the support of families who understand their needs on a personal level.

## 6. Teach Families What to Expect on a Weekly Basis

Collaborative customized care plans need to be coordinated well before discharge [9]. Gaining an understanding of the weekly and daily tasks for the baby can help a family in their planning. For example, many families do not realize that a child with a G-tube cannot enroll in traditional childcare and that alternate arrangements must be made. It is important for families to be able to ask questions like: What will a typical week look like? What appointments should I expect and when? Is there a case-worker assigned to help navigate insurance claims and for ad hoc support? What additional supports are needed at home? Who will care for the baby? What training is needed? When to call to order medical supplies so you do not run out of oxygen? How to manage pets when there are likely allergy risks or if your pet likes to chew on cords such as oxygen cords? How can we put the appropriate safety measures in place? Will my baby develop normally? Will my baby have a setback? Will we notice warning signs of a change in condition and have time to intervene? How will we manage without monitors? Will we be followed by our regular pediatrician? Will we have the additional support we need? Families are overwhelmed and need to feel supported as they navigate through their new normal.

## 7. Support Family Well-Being

While not all babies in the NICU are a very low birthweight, there are additional unknowns and considerations to be aware of to care for a medically complex child. These children are likely to see several specialists and many will be monitored by the hospital’s high-risk follow-up clinic in addition to both private and public community-based early intervention programs. Many of the various medical and developmental support services do not communicate well with each other. The parents become the people to ensure all parties are on the same page and communicate key information to each provider. The parents are the coordinators for all of these entities while carrying the weight of the challenges they face as a parent of a child with special needs. If the family becomes overwhelmed, it can be more difficult for them to provide quality care for their child. For this reason, follow-up clinics should not solely focus on the baby’s development. They must also consider the family’s well-being as essential to providing support and care. A more holistic approach could include making family support services available to families to address social determinants of health before or after their clinic appointments and the family’s concerns at that time.

Relationships between families and providers take time to establish trust and rapport. While we have been focusing on general experiences, we must also acknowledge that disparities are prevalent. Black, Hispanic and Native American families are less likely to be referred to an appropriate high-risk NICU follow-up program [10]. Structural racism must be addressed to reduce health disparities. Clinics could engage community-based organizations who best know the resources and supports available in that demographic and also provide pre-discharge consultations inside the unit itself. Some programs are designed to focus on emotional health and resilience-building capacities. Other programs are focused on educational needs such as explaining the benefits of skin-to-skin contact. A holistic approach must have some built-in flexibility to address the unique needs of each family.

## 8. Co-Create a Care Plan with a Family-Centered Design

It is important to co-develop care plans with families that reflect the family’s priorities and decisions for care after discharge [11]. Some teams build a flight plan to assist families so that they know which questions to ask during the NICU stay. A flight plan is a navigation tool that can help families find care and support and identify community resources after discharge. Each family should not be forced to piece together a care plan as if no soul had ever been down this road before. Families also need emotional support, educational support, sibling support, and assistance in identifying resources. The plan must be communicated not just verbally but through multiple formats; i.e., visuals and narrative. Providers need to organize the critical information for families to assist them in navigating the system and to help them connect with the appropriate providers. Creating worksheets or journals to guide families to document daily schedules, medication times, the use of food logs, liquid logs, and appointment trackers are tools that can greatly improve the family’s quality of life, improve efficiency, and reduce stress.

## 9. Use Technology to Assist with Transition

In the first 2 years after discharge, it is not unusual to connect with over 20 providers/specialists, averaging 5 appointments a week. It would be helpful to have guidance on expected future needs so that families can anticipate care needs, identify viable options, and be placed on waitlists as needed. Trying to process and retain information while under significant emotional strain is like treading water. Families would greatly benefit from a simple tracker for upcoming appointments; for example, cardio in 6 months, pulmonology in 3 months. It can be difficult to keep track of all appointments and care needs after discharge. Some families may miss appointments and are then never connected with needed services or become overwhelmed with secondary strains. There are several apps and technological platforms that have been developed to reduce the burden on families and ensure that all families can access services and support [12,13,14]. For some families, it is not possible to maintain full-time employment and attend five–seven appointments per week. Finding financial support for families who are losing income as the result of the NICU experience may be a challenge, but it must be a part of the discussion. Telehealth appointments are also a great way to relieve the burden of multiple appointments, and free up more time for families who may otherwise need to travel for several hours to consult with a specialist.

## 10. Avoid Trauma Triggers

Medical appointments can trigger trauma for NICU families, causing them to “re-live” their NICU experience and recall distressing events. The potential for trauma may be increased by the way information is presented or by the need for families to recite their child’s full medical history at the start of each appointment. Not only is this both physically and mentally exhausting, it forces the family to recall the details of traumatic events on a regular, reoccurring basis. Electronic medical record data can be a great tool for sharing patient data with multiple providers. A summary page for each provider to see the key points could benefit all involved. Parents should bring a list of medications and dosages to every appointment so they do not have to rely on memory. Although providers take the time to share information with families, it may not be retained or understood. A one-page summary in layman’s terms describing each condition, each surgery, and key takeaways would be helpful to document. These could be written as brief bullets not embedded in the lengthy medical files or discharge paperwork.

## 11. Prepare Families for What to Do in an Emergency

This one-page summary can be critically important and even lifesaving in an emergency. Preparing families for emergency room (ER) visits post-discharge can be challenging but knowing that emergency intervention may be needed is helpful to set expectations and mentally prepare families [15]. Emergency situations can escalate fast. Knowing the warning signs of when to react by calling 911 or going to the ER requires acute awareness of your child’s baseline behaviors and emergent needs. From Jessica’s perspective: My son tallied a total of 1 month of inpatient days after NICU discharge. Parents can reach out to a medical professional to determine if an ER visit is recommended. Both parents may not agree on when an ER visit is necessary, so it is important to have an expert consult on call. From LaToshia’s perspective: I had been taught by the nurses how to tell when my baby stopped breathing without the machines. The nurse allowed me to assess breathing during feedings because that was typically when my son would drop his saturations. I was able to tell my son was not breathing before the machine would beep. That to me was what I needed to give me the confidence to take my son home without the machines. Once we were home, he did have breathing difficulty. I was able to spot it and bring my son to the ER with the information needed to expedite care. The NICU nurse perceived my fears related to breathing and recognized a learning opportunity that would prepare me for ER decisions. This was key to saving my son’s life after discharge. A written emergency plan may be helpful to assist families in stressful moments. Who do you call when X happens? A list of important numbers including a 24-7 support-nurse line should be included. The plan must include important practical details that, if not addressed, could delay the receipt of care. For example, securing emergency care for siblings when you are running to the ER. Who in the community can you consult in an emergency? It is important to include in the written emergency plan a list of neighborhood contacts and supports as well.

## 12. Recommendations

The following recommendations offer opportunities to support families as they transition home (Table 1).

## 13. Conclusions

The healthcare system can be challenging for families to navigate. Many families are not sure where to go for their needs and they become the default case manager and connection point for all the specialists their baby will see. The current fragmented system of care needs to be pieced together by the parent, often resulting in gaps in needed services that must be recognized and adapted to. Some families would greatly benefit from the support of someone with expertise, like a patient navigator, to assist in navigating the health system, local care options, and in identifying resources. Coordinating care is incredibly time intensive and logistically challenging. We need to empower families to better navigate the systems of care and to anticipate care needs due to long waitlists. Families need an advocate to ensure their child receives their recommended therapies and early interventions and an easy-to-follow schedule to survive this intensive period of clinical encounters. With support, families and their NICU babies can thrive through adversity and experience a successful transition to the home that sets them on a path of positive health and well-being across the life course.

## Figures and Tables

**Table 1:** **Recommendations for the transition home post-NICU discharge.**

Give parents and caregivers an opportunity to share their hopes and concerns during rounds prior to staff updates. Seek various opportunities to engage families who are not present during rounds. Develop a collaborative care model as a protective factor for families transitioning home. Engage parents and caregivers in routine tasks (e.g., weighing the baby, diaper changes, G-tube flushes, etc.) so that they feel confident performing these tasks independently after they transition home. Teach families how to perceive warning signs without machines to build confidence. Address structural racism in the NICU to reduce health disparities [16]. Prepare a flight plan for families to understand what happens at each stage of discharge. Connect families with community support and advocacy organizations. Help families to plan for the transition home. Some considerations include: in-home support, equipment, work schedules, sibling needs, financial needs, community resources, peer support, preparing for the follow-up clinic, and plans for siblings if an ER visit is needed. Screen for social determinants of health (SDOH) regularly during pediatric visits. Develop a summary page from each medical encounter to communicate critical information to multiple providers and families. Provide a printable care plan for families to take home after various appointments to reduce the burden of relying on memory. Use technological platforms to improve care coordination and reduce the burden on families.

## Data Availability

Not applicable.
